# The Four Horsemen in Colon Cancer

**DOI:** 10.1155/2019/5636272

**Published:** 2019-09-29

**Authors:** Marco Antonio Hernández-Luna, Sergio López-Briones, Rosendo Luria-Pérez

**Affiliations:** ^1^Department of Medicine and Nutrition, University of Guanajuato, Campus Leon, Leon, Guanajuato, Mexico; ^2^Unit of Investigative Research on Oncological Diseases, Children's Hospital of Mexico Federico Gomez, Mexico City, Mexico

## Abstract

Worldwide, neoplasms of the gastrointestinal tract have a very high incidence and mortality. Among these, colorectal cancer, which includes colon and rectum malignancies, representing both highest incidence and mortality. While gallbladder cancer, another neoplasm associated to gastrointestinal tract occurs less frequently. Genetic factors, inflammation and nutrition are important risk factors associated with colorectal cancer development. Likewise, pathogenic microorganisms inducing intestinal dysbiosis have become an important scope to determine the role of bacterial infection on tumorigenesis. Interestingly, in human biopsies of different types of gastrointestinal tract cancer, the presence of different bacterial strains, such as *Fusobacterium nucleatum*, *Escherichia coli*, *Bacteroides fragilis* and *Salmonella enterica* have been detected, and it has been considered as a high-risk factor to cancer development. Therefore, pathogens infection could contribute to neoplastic development through different mechanisms; including intestinal dysbiosis, inflammation, evasion of tumoral immune response and activation of pro-tumoral signaling pathways, such as *β* catenin. Here, we have reviewed the suggested bacterial molecular mechanisms and their possible role on development and progression of gastrointestinal neoplasms, focusing mainly on colon neoplasms, where the bacteria *Fusobacterium nucleatum*, *Escherichia coli*, *Bacteroides fragilis* and *Salmonella enterica* infect.

## 1. Introduction

Worldwide, neoplasms affecting gastrointestinal tract are among the most frequent in incidence and mortality [1]. Gastrointestinal tract neoplasms are including: colon, rectum, stomach, pancreas, biliary tract and esophagus [2]. The main factors associated with development of gastrointestinal tract malignancies are alcohol consumption and smoking [3–5], high fat diets [6–9]; as well as, ageing, gender and race [10–13]. In addition, pathogenic microorganisms such as viruses and bacteria infecting the gastrointestinal tract, are being studied as possible triggers for development of neoplasms. In this regard, the role of *Helicobacter pylori* in the development of gastric cancer has been extensively studied [14]. However, other bacteria have also been associated with development of gastrointestinal neoplasms, especially in colon, rectum and gallbladder. This review describes the possible roles of *Fusobacterium nucleatum*, *Escherichia coli*, *Bacteroides fragilis* and *Salmonella enterica* on cancer development. These bacteria have been considered as emerging pathogenic bacteria associated with development of colorectal cancer, which includes colon and rectum neoplasms, [15]. Here, we have focused on colon cancer, a neoplasia with a very high incidence on worldwide population, registering in 2018; 850,000 new cases and a mortality rate of 550,000 individuals [1].

## 2. *Fusobacterium nucleatum*

*Fusobacterium nucleatum* (*F. nucleatum*) is an adherent and invasive Gram-negative anaerobic bacterium. *F. nucleatum* resides mainly in oral cavity and is usually associated with periodontal disease [16]. Nevertheless, in last years, this bacterium has been detected in primary lesions [17], biopsies [18, 19], and stools [20] of patients with colon cancer, so bacterium has also been linked to development and progression of this neoplasia. In addition, different regions of human colon are colonized by *F. nucleatum* [21]. However, in patients with colon cancer, *F. nucleatum* has been located mainly on cecum and rectum [22, 23], where it is preferentially localized into tumor tissue [24, 25]. An important factor associated with *F. nucleatum* recruitment into tumor is over-expression of Gal-GalNAc molecules by tumor cells, which promote bacterial adhesion via Fap2 protein [26]. Likewise, high levels of anti-*Fusobacterium* IgA and IgG antibodies have been detected in sera of colon cancer patients [27], which could be used as biomarkers in early diagnosis of this neoplasia. Additionally, infection by *F. nucleatum* has been associated with a low survival of colon cancer patients [28], as well as increased resistance to chemotherapy treatment [29].

Previous studies have reported the association of *F. nucleatum* and colon cancer, although the presence of this bacterium in infected people is highly variable and inconsistent. In this regard, infection with *F. nucleatum* has been detected in 15% of North American population with colon cancer, while more than 60% of infected patients have been found in Chinese population [25, 28, 30, 31]. Interestingly, common characteristics found in all colon cancer patients with *F. nucleatum* infection were microsatellite instability (MSI), methylation phenotype of CpG island (CIMP), as well as *BRAF* and *KRAS* genes mutations [23, 25, 32].

On the other hand, infection with *F. nucleatum* in C57BL/6 APC^Min/+^ mice induced tumorigenesis regardless of colitis development [20], unlike the infection by enterotoxigenic *Bacteroides fragilis*, which initially produces colitis and subsequently tumors [33]. Therefore, several mechanisms inducing tumor by *F. nucleatum* have been proposed, including *β* catenin signaling pathway activation, which is upregulated in colon cancer [34]. In this pathway, *β* catenin is phosphorylated by PAK-1 through *F. nucleatum*-TLR4 interaction [35]. Likewise, binding of *F. nucleatum* FadA adhesin to E-cadherin expressed on host cells activates the Wnt/*β* catenin pathway promoting cell proliferation [36]. Additionally, a significant decrease on expression of TOX family proteins (thymocyte selection-associated high-mobility group box) after *F. nucleatum* infection has been shown [37]. These proteins regulate important cellular functions such as growth, apoptosis, DNA repair and metastatic processes [38]. Interestingly, an important decrease on TOX family proteins expression has been associated with advanced tumors.

Another mechanism associated with development and progression of colon cancer induced by *F. nucleatum* have been linked to inflammation. Thus, in colon cancer patients infected with *F. nucleatum*, an important increase on TNF-*α* and IL-10 expression levels have been shown in adenomas, a precursor lesion of colon cancer [17]; while into tumor, IL-6 and IL-8 increased levels were induced by *F. nucleatum*. Both IL-6 and IL-8 are proinflammatory cytokines regulated by NF-κB transcription factor, a link between inflammation and cancer; and NF-κB activation has been also shown in colon cancer [18, 36]. Additionally, *F. nucleatum* infection increased the chemokine CCL20 expression [39], a chemokine related with both colon cancer progression [40], and Th17 + lymphocytes mediated inflammatory response [41]. Likewise, *F. nucleatum* induced inflammation could be regulated by microRNAs, such as miR-135b; because a correlation between *F. nucleatum* and miR-135b overexpression in colon cancer patients has been found [42]. So it has suggested that miR-135b could also be used as a biomarker in early detection of colon cancer [43]. However, the role of *F. nucleatum* in development and progression of colon cancer remains to be understood.

**Figure 1:**
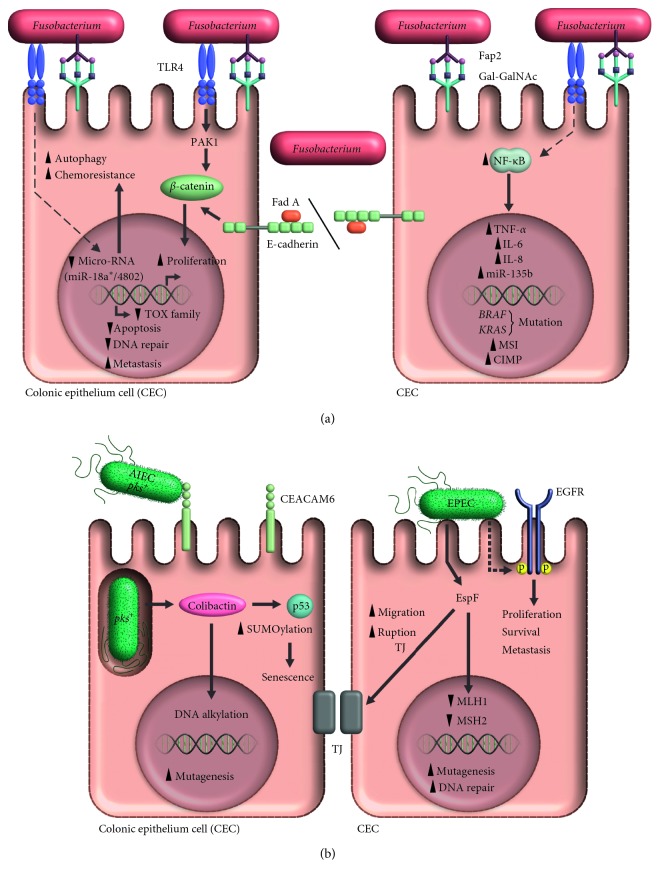
Oncogenic activity of *Fusobacterium nucleatum* and *Escherichia coli.* (a) Gal-GalNAc overexpression in colon cells promotes the recruitment of *Fusobacterium nucleatum* via the Fap2 protein. After interacting with TLR4, the bacterium activates the protein PAK 1 and in turn, *β* catenin; the latter can also be activated through the effect of FadA on E-Cadherin. Activation of these signaling pathways promotes cellular proliferation and decreases proteins of the TOX family, which are associated with decreased apoptosis, failures in DNA repair and increased metastases. Likewise, bacterial interaction with TLR4 and its signaling via MYD88, modulates specific microRNAs that activate the autophagy associated with chemotherapy resistance. Also, *Fusobacterium nucleatum* increases the inflammatory process characterized by the presence of cytokines such as TNF-*α*, IL-6 and IL-8, that are regulated by the transcription factor NF-κB, whose increased activation has also been documented in colon cancer. *Fusobacterium nucleatum* has also been shown to be associated with the development of mutations in the genes *BRAF* and *KRAS*, microsatellite instability (MSI) and the methylation phenotype in CpG islands (CIMP). (b). The Adherent Invasive *Escherichia coli* strain (AIEC) colonizes the intestinal epithelium and uses CEACAM6 to invade the cells of the colonic epithelium; once internalized, it produces colibactin, a cyclomodulin encoded by the *pks* island, that damages DNA by alkylation and promotes the development of mutations. Colibactin also fosters cellular senescence by favoring SUMOylation of p53. Infection with the Enteropathogenic *Escherichia coli* (EPEC) strain, promotes the autophosphorylation of EGFR, a protein associated with an increase in proliferation, survival and metastases; it also decreases the expression of the DNA repair proteins, MLH1 and MSH2, and favors the rupture of tight junctions, a process involved in the development of metastases. All these EPEC-dependent mechanisms have been associated with the EspF protein.

Finally, microsatellite instability (MSI) in colon cancer has been linked to capability to evade immune response by *F. nucleatum* infected tumor cells [31]. In this fact, CD3+ [32], and T CD4+ lymphocytes subsets were decreased into the tumor after *F. nucleatum* infection [37], but proportions of T CD8+, CD45RO+, or FOXP3+ lymphocytes subsets were not modified [32]. In addition, the binding of *F. nucleatum* Fap2 protein with TIGIT [44], a receptor with tyrosine-based inhibitory motif (ITIM) expressed on NK cells [45], leads to an important decreased on lymphocytes infiltration into tumor. This way, tumor is protected from an effective immune cells attack [44]. The proposed mechanisms are summarized in Figure 1(a).

## 3. *Escherichia coli*

*Escherichia coli* (*E. coli*) is a Gram-negative bacterium widely distributed in nature, including human intestinal microbiome. The *E. coli* strains are classified into 5 phylogenetic groups: A, B1, B2, D, and E [46]. The main *E. coli* strains associated with human disease belong to B2 group and are also related to colon cancer [47, 48]. To date, the role of pathogenic *E. coli* strains in carcinogenesis is not completely known; however, chronic inflammation in gastrointestinal tract that they promote has been suggested as the trigger mechanism [49]. Because, this chronic inflammation induces pathologies such as Crohn's disease [50], an important risk factor to develop colon cancer [51]. Alternatively, molecular mechanisms induced directly by bacteria have been described. *In vitro* studies have shown that pathogenic strains such as Adherent-Invasive *Escherichia coli* (AIEC) and Enteropathogenic *Escherichia coli* (EPEC), secrete cyclomodulin colibactin [52] and effector protein EspF [53], respectively, which are involved in development and progression of colon cancer. Although the specific mechanisms associated to colon cancer induced by pathogenic *E. coli* have started to become elucidated recently. The molecular mechanisms associated to colon cancer and pathogenic *E. coli* are described in Figure 1(b).

### 3.1. Adherent-Invasive *Escherichia coli*

The main pathogenic *E. coli* strain found in tumor tissue from colon cancer patients is Adherent-Invasive *Escherichia coli* or AIEC [54]. On infection, AIEC binds to CEACAM6 (cellular adhesion receptor associated to carcinoembryonic antigen) [55], which is overexpressed on intestinal epithelial cells of both Crohn's disease and colon cancer patients [56]. To date, it is still unknown what induces overexpression of CEACAM6 on the intestinal epithelium in these patients, although it has been shown that IL-6 is related to induction of CEACAM6 expression [57]. Additionally, it well is known that infection with AIEC stimulates IL-6 production [58]. Taking all these finding together, it is suggested that AIEC could regulate its own infective capacity on intestinal epithelium by both increasing IL-6 production and CEACAM6 expression, and when bacterium has penetrated and invaded the intestinal epithelium, carcinogenesis could be induced through secretion of colibactin, although the true mechanism is not completely known.

### 3.2. Colibactin and the *pks* Island

Colibactin is a cyclomodulin encoded in the genotoxic *pks* island (polyketide island). The *pks* island has been found in different *E. coli* strains [59, 60]. Colibactin is a secondary metabolite produced by non-ribosomal peptide synthetase (NRPS)–polyketide synthase (PKS) (NRPS-PKS). Although the synthesis of colibactin is not completely known, it has been shown that a multi-enzymatic complex is required in which several genes of *pks* island participate [61, 62]. The main role of colibactin in carcinogenesis has been associated with DNA damage [63], by acting as an alkylating agent [64, 65], inducing DNA mutations and promoting tumor development.

On the other hand, because of the synthesis of colibactin has not yet been achieved, which has prevented the understanding of the molecular mechanism of this cyclomodulin, most studies designed to evaluate the role of colibactin in carcinogenesis have been limited to study the *pks* island function. *In vitro* infection of cell lines with *E. coli pks* + strains induced a cell cycle arrest, aneuploidy and tetraploidy [66, 67]; as well as, cell senescence via miR-20a-5P, which inhibits the expression of SUMO-specific protease 1 (SENP-1) [52]. SENP-1 is a protein that induce deSUMOylation of p53 [68], an important transcription factor involved in regulation of cellular senescence and development of colon cancer [69]. On the other hand, the role of the *pks* island has been evaluated in experimental murine models. The inflammatory environment in mice intestinal epithelium induced upon infection, both spreading of *E. coli pks*+ and increased risk of colon cancer were produced [49, 70]. In a xenotransplant murine model, infection with *E. coli pks* *+* strains lead to a significant increase in tumor size, while infection with *E. coli pks–*strains do not [52].

### 3.3. Enteropathogenic *Escherichia coli*

Enteropathogenic *Escherichia coli* or EPEC, is the second pathogenic strain of *E. coli* associated to colon cancer [71, 72], and it has been suggested that EPEC infection might be involved in some molecular pathways involved in colorectal tumorigenesis [72]. *In vitro* studies have shown that infection with EPEC stimulates macrophage-inhibitory cytokine-1 (MIC-1) production, a cytokine related to metastasis by inducing both, increasing survival and spreading of tumor cells through a GTPase Rho A-dependent pathway [73]. Likewise, autophosphorylation of EGFR receptor, was induced upon EPEC infection [74]; this is a upstream activator of both prosurvival phosphoinositide 3-kinase/Akt and proinflammatory mitogen-activated protein (MAP) kinase pathways. These molecular mechanisms have been associated with colon cancer [75], and poor prognosis in patients [76].

However, it has been shown that EPEC can degrade EGFR receptor via EspF protein [77]; this effector protein is internalized to epithelial cells through the *E. coli* type III secretion system [78]. Interestingly, this process can be inhibited by EspZ, another protein that is also internalized into epithelial cell through the same secretion system [77]. On the other hand, EspF has also been associated with other mechanism inducing cancer, such as decreasing levels of DNA repair proteins MLH1 and MSH2 (mismatch repair MMR) [53, 71], which are widely related to colon cancer [79]. Further, EspF could also contribute to colon cancer metastasis by promoting detachment and dissemination of tumor cells through rupturing tight junction proteins such as Occludin and Claudin on intestinal epithelium [80].

Finally, other proteins produced by pathogenic *E. coli* strains and related to carcinogenesis have been studied. These proteins include: (1) *Cytolethal distending toxin* (CDT), which blocks cell cycle [81], and induces malignant transformation of epithelial cells [82], (2) *Cycle inhibiting factor* (Cif), which induces nuclear DNA elongation on cells and stimulates DNA synthesis even when infected cells are not actively dividing [83] and (3) *Cytotoxic Necrotizing Factor 1* (CNF1), which induces gene transcription and cellular proliferation by GTPases activation [84].

## 4. *Bacteroides fragilis*

The *bacteroides* is a normal inhabitant of human intestine and represent about 30% of intestinal microbiota [85]. These bacteria have a very important role on mucosal immune system development [86], and intestinal homeostasis [87]*. Bacteroides fragilis* (*B. fragilis*) is classified within *bacteroides* species and is an anaerobic Gram-negative bacterium colonizing about 0.5% to 2% of whole human intestine [86, 88, 89]. Two *Bacteroides fragilis* strain has been described: (a) non-toxigenic *B. fragilis* or NTBF and (b) toxigenic *B. fragilis* or ETBF, which is characterized by a 6 kb pathogenicity island encoding to a metalloproteinase, also known as *B. fragilis* toxin (BFT) or fragilysin [90], of which 3 isoforms have been identified [91].

It has been shown that while NTBF has a protective effect against the development of colitis and colon cancer [92], ETBF has been associated with a wide variety of clinical manifestations ranging from a simple diarrhea to inflammatory bowel disease and colitis [93], both considered as high-risk factors to develop colon cancer. ETBF has already been associated to colon cancer [88], because bacteria has been detected in stool and biopsies obtained from colon cancer patients [94], particularly in early cancer stages [95]. However, a very low proportion of ETBF has been detected in stools from healthy individuals [96].

Although role of enterotoxigenic *B. fragilis* in development of colon cancer has not been completely described; different studies have shown that carcinogenesis induced by ETBF is through BFT toxin, which is present in ETBF but not in NTBF bacteria strains. BTF toxin is a multifunctional protein; thus, it could induce to tumorigenesis through several mechanisms including activation of c-Myc [97], and consequently an increase on spermine oxidase (SMO) expression [98], an enzyme increasing reactive oxygen species (ROS), which favors cellular injury and carcinogenesis.

Another possible mechanism of ETBF toxin-mediated carcinogenesis, could be through host immune system dysregulation, inducing the recruitment and accumulation of Treg lymphocytes in intestinal lamina in response to bacteria [99], which subsequently suppress the mucosal Th1 response and polarizing to Th17 lymphocytes response [100] by increasing IL-17 secretion [33]. Interestingly, increased levels of IL-17 have been detected on early weeks post-infection, after that; its expression was decreased. However, in APC^Min/+^ mice, the early and temporary increased on IL-17 was enough to trigger tumorigenesis [101]. On this regards, it has been suggested that activation of Stat3 [102] and NF-κB [103] pathways by immune responding cells and colonic epithelial cells (CECs) may be involved [104]. Furthermore, ETBF also polarizes IL-17-secreting TCR*γδ*+ T lymphocytes [105], promoting the differentiation and recruitment of myeloid-derived suppressor cells (MDSC) into the tumor [106, 107], which has been associated with a poor prognosis of colon cancer patients [108]. Because IL-17 up regulates CXCL1, CXCL2 and CXCL5 chemokines expression, also has been involved on MDSC recruitment [104]. Additionally, T lymphocyte proliferation is inhibited by high levels of Nitric Oxide (NO), and arginase 1 (Arg1) a potent metabolic enzyme induced and produced by an increase on MDSC population [107], this way several mechanisms of evasion of anti-tumor immune response by tumor cells are generated.

**Figure 2:**
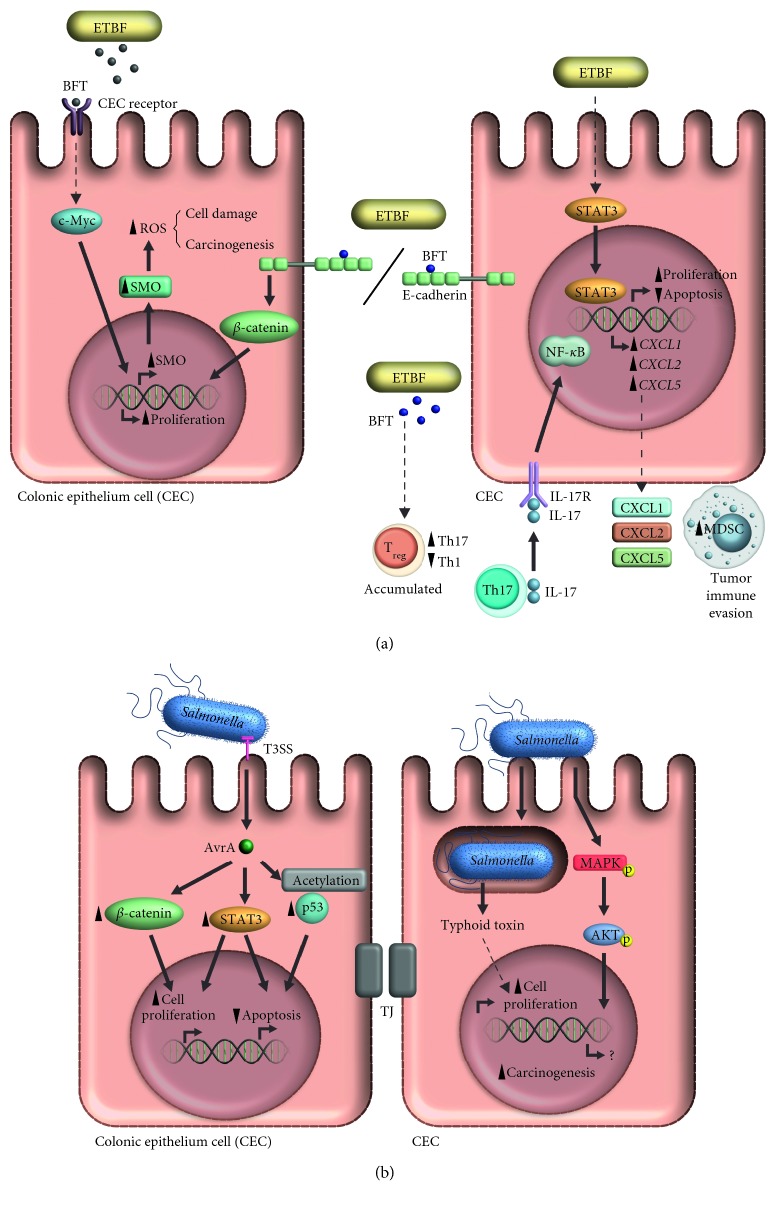
Oncogenic activity of *Bacteroides fragilis* and *Salmonella enterica*. (a) Enterotoxigenic *Bacteroides fragilis* (ETBF) stimulates carcinogenesis in colonic epithelium through the BFT toxin. This toxin leads to an increase in reactive oxygen species (ROS) by inducing spermine oxidase expression via c-Myc. Likewise, BFT cuts E-cadherin, thus activating *β* catenin which stimulates cellular proliferation. BFT also modulates the host's immune response by promoting Treg lymphocytes to polarize the response to Th17 lymphocytes, thus increasing IL-17 secretion which in turn, activates NF-κB in the colonic epithelium; this results in the secretion of the chemokines CXCL1, CXCL2 and CXCL5 that recruit MDSC, thus favoring evasion from the immune response. The presence of ETBF has also been associated with STAT3 activation. (b). *Salmonella enterica* releases two proteins that promote carcinogenesis: the typhoid toxin that induces cellular proliferation, and the AvrA protein that is internalized via the Type 3 Secretion System(T3SS). AvrA activates the *β* catenin and STAT3 pathways, and also causes the acetylation of p53. Additionally, *Salmonella enterica* leads to the activation of the MAPK/AKT pathway. The activation of these pathways promotes an increase in proliferation and cellular differentiation, and decreases apoptosis.

Finally, ETBF could trigger carcinogenesis through *β* catenin pathway activation, by disrupting the adherent E-cadherin gap junctions, similar than *F. nucleatum*, [109, 110]. The molecular carcinogenic mechanisms of ETBF are summarized in Figure 2(a).

## 5. *Salmonella enterica*

*Salmonella* enterica represents a broad range of bacteria, including serotypes such as *Salmonella* Typhi (*S.* Typhi), *Salmonella* Paratyphi (*S.* Paratyphi), *Salmonella* Enteritidis (*S*. Enteritidis) and *Salmonella* Typhimurium (*S.* Typhimurium) [111]. In recent years, development of colon cancer [112], gallbladder cancer [113], and other gastrointestinal tract neoplasms have been associated with *Salmonella enterica* infection. Also, It has been found that bacteria may modulate host immune response [114], promoting carcinogenesis by both DNA damage and increasing proliferation, as well as cell migration through induction of chronic inflammation [115]. At least, two proteins of *Salmonella enterica* have been associated with an increased risk of developing colon cancer. The former is typhoid toxin; a cyclomodulin sharing features with the *E*. *coli* CDT [116], increasing cellular survival and promoting intestinal dysbiosis [117]. Both mechanisms are involved with development of inflammatory bowel disease and colon cancer [118]. The second protein is AvrA, an effector protein secreted by bacteria through type III Secretion System [119], and it has been detected in stool samples from colon cancer patients [120].

Thus, the main protein of *Salmonella enterica* associated with carcinogenesis is AvrA. It has been suggested that most important role of AvrA in colon cancer may be related to inflammatory and immune response dysregulation, through several mechanisms such as: inhibition of NF-_Κ_B signaling pathway [121], inhibition of IL-12, INF-*γ* and TNF-*α* secretion [122], inhibition of IL-6 transcription and increasing on IL-10 transcription [123]. On the other hand, AvrA has been associated to tumors on intestinal epithelium through activation of Wnt/*β* catenin, inducing cellular proliferation [124], by both *β* catenin phosphorylation (activation) and deubiquitination (decreased degradation) [125]. These mechanism are important in signaling pathway associated with colon cancer development [126]. Likewise, JAK/STAT signaling pathway is activated by AvrA [127], which regulates several mechanisms such as: apoptosis, cellular proliferation and differentiation, as well as inflammatory response, all these important events involved in carcinogenesis [128]. Additionally, the function of p53 transcription factor is affected by AvrA acetyl transferase activity [129], leading to cell cycle arrest and inhibition of apoptosis by decreasing pro-apoptotic proteins (such as Bax), dependent of p53 acetylation [130]. The carcinogenic mechanisms associated to *Salmonella enterica* are summarized in Figure 2(b).

### 5.1. Salmonella enterica and Gallbladder Cancer

Gallbladder cancer is the main type of neoplasia affecting the biliary tract. Worldwide, the incidence of this neoplasia is low. Interestingly, it has been shown that gallbladder cancer occurs more frequently in geographic regions with a high incidence of *Salmonella* infection [113, 131–134]. Therefore, a greater interest has been generated in searching for a possible association between *Salmonella* infection and development of gallbladder cancer. On this respect, Typhoidal *Salmonella* serotypes as *S.* Typhi and *S*. Paratyphi have been detected in most of the biopsies from patients with gallbladder cancer [113, 135–137], however, DNA traces of Non-typhoidal *Salmonella* serotypes as *S.* Typhimurium and *S.* Choleraesuis have also been found in gallbladder cancer biopsies [135]. These findings have suggested that *Salmonella* (which may be undetected for years, because it can produce biofilm on cholesterol biliary stones [138]), could represent an important risk factor in development of gallbladder cancer [132], because inflammation and epithelial injury associated to cholelithiasis is induced by *Salmonella* [139] and cholelithiasis is a common clinical manifestation in most patients with gallbladder cancer [137]. However, the mechanism triggering carcinogenesis by *Salmonella enterica* in gallbladder is not completely known, but it has been suggested that a chronic inflammation of gallbladder is induced [140], after bacteria arrival to gallbladder from either blood circulation or bile [141].

Additionally, recruitment of some immune cells, including activated macrophages expressing COX-2 is increased upon *Salmonella enterica* infection [142]. COX-2 is an important enzyme that promotes the development of gastrointestinal tract tumors [143, 144]. Also, bacteria induced inflammation leads to mutations of *TP53* gene, increasing the risk of developing gallbladder cancer [145]. Finally, *in vitro* infection of cell lines and gallbladder organoids with *S.* Typhimurium, led to malignant transformation though MAPK and AKT signaling pathways activation. Similarly, *in vivo* activation of these signaling pathways resulted in tumor development in mice [134].

## 6. Conclusions

Recently, the number of publications referring an association between pathogenic bacteria and development of gastrointestinal tumors, has increased exponentially. The best example and widely reported is *Helicobacter pylori* and gastric cancer. However, emerging bacteria such as *Fusobacterium nucleatum*, *Escherichia coli*, *Bacteroides fragilis* and *Salmonella enterica* have also been involved in development of cancer, particularly colon cancer.

In this review, it is suggested that infection by pathogenic bacteria may be a high-risk factor associated with the development of neoplasms in gastrointestinal tract. Mechanisms such as, inflammation, modulation and evasion of immune response and activation of signaling pathways, such as the *β*-catenin pathway; all are potential triggers of carcinogenesis.

The inducing tumor mechanisms can be evaluated in murine models, such as APC^Min/+^, a specific mice model to study intestinal tumorigenesis [146]. In this experimental model, developing colon cancer mechanisms by *Fusobacterium nucleatum*, *Escherichia coli*, *Bacteroides fragilis* and *Salmonella enterica* have been identified. However, effects of coinfection with these bacteria and tumor development remains to be analyzed, because ETBF and *E. coli pks* + strains have been found simultaneously in patients with adenomatous polyps, a precursor lesion of colon cancer [147]. Nevertheless, ETBF is a very common bacterium in colon cancer patients but also in healthy individuals [96], so it remains to be elucidated whether ETBF has a role on induction of carcinogenesis. Another possible mechanism through bacteria may trigger cancer is by biofilm. This structure produced by a community of bacteria, more common in ascending colon [148], could increases carcinogenic metabolites concentration, such as polyamines [149], which are related to an important increase on reactive oxygen species. In addition, biofilm has been associated with decreased expression of E-cadherin on colonic epithelial cell, an over activation of IL-6 and Stat3 in epithelial cell [148], all these mechanisms are involved in colon cancer. The mechanisms above described, are used by *Fusobacterium nucleatum*, *Escherichia coli*, *Bacteroides fragilis* and *Salmonella enterica*. Therefore, further studies are required to understand the specific roles of these four bacteria in development of neoplasms on gastrointestinal tract, specifically in colon cancer.

## 7. Future Perspectives

Worldwide, colon cancer has very high incidence and mortality. Here we have described that infection with either bacteria such as *F. nucleatum*, *E. coli*, *B. fragilis* or *S. enterica* represent an important risk factor that promote cell transformation (carcinogenesis). In this regards, detection of promoting carcinogenesis bacterial proteins, such as cyclomodulin, colibactin, BFT, AvrA or EspF may be used as a biomarker for early detection of colon cancer, as it has been proposed for Fap2 [150]. Because early detection of tumor can increase both healing and survival. Moreover, it would generate new and appropriate strategies to block bacterial proteins activity, thus complementing the traditional treatment to neoplasms of gastrointestinal tract.

## References

[1] Bray F., Ferlay J., Soerjomataram I., Siegel R. L., Torre L. A., Jemal A. (2018). Global cancer statistics 2018: GLOBOCAN estimates of incidence and mortality worldwide for 36 cancers in 185 countries. *CA: A Cancer Journal for Clinicians*.

[2] Bijlsma M. F., Sadanandam A., Tan P., Vermeulen L. (2017). Molecular subtypes in cancers of the gastrointestinal tract. *Nature Reviews Gastroenterology & Hepatology*.

[3] Park S.-Y., Wilkens L. R., Setiawan V. W., Monroe K. R., Haiman C. A., Le Marchand L. (2019). Alcohol intake and colorectal cancer risk in the multiethnic cohort study. *American Journal of Epidemiology*.

[4] Vanella G., Archibugi L., Stigliano S., Capurso G. (2019). Alcohol and gastrointestinal cancers. *Current Opinion in Gastroenterology*.

[5] Fagunwa I. O., Loughrey M. B., Coleman H. G. (2017). Alcohol, smoking and the risk of premalignant and malignant colorectal neoplasms. *Best Practice & Research Clinical Gastroenterology*.

[6] Triff K., McLean M. W., Callaway E., Goldsby J., Ivanov I., Chapkin R. S. (2018). Dietary fat and fiber interact to uniquely modify global histone post-translational epigenetic programming in a rat colon cancer progression model. *International Journal of Cancer*.

[7] Nadella S., Burks J., Al-Sabban A. (2018). Dietary fat stimulates pancreatic cancer growth and promotes fibrosis of the tumor microenvironment through the cholecystokinin receptor. *American Journal of Physiology-Gastrointestinal and Liver Physiology*.

[8] Arita S., Kinoshita Y., Ushida K., Enomoto A., Inagaki-Ohara K. (2016). High-fat diet feeding promotes stemness and precancerous changes in murine gastric mucosa mediated by leptin receptor signaling pathway. *Archives of Biochemistry and Biophysics*.

[9] Fowler A. J., Richer A. L., Bremner R. M., Inge L. J. (2015). A high-fat diet is associated with altered adipokine production and a more aggressive esophageal adenocarcinoma phenotype in vivo. *The Journal of Thoracic and Cardiovascular Surgery*.

[10] Tramontano A. C., Nipp R., Mercaldo N. D., Kong C. Y., Schrag D., Hur C. (2018). Survival disparities by race and ethnicity in early esophageal cancer. *Digestive Diseases and Sciences*.

[11] Ashktorab H., Kupfer S. S., Brim H., Carethers J. M. (2017). Racial disparity in gastrointestinal cancer risk. *Gastroenterology*.

[12] Luo G., Zhang Y., Guo P., Wang L., Huang Y., Li K. (2017). Global patterns and trends in stomach cancer incidence: age, period and birth cohort analysis. *International Journal of Cancer*.

[13] Øines M., Helsingen L. M., Bretthauer M., Emilsson L. (2017). Epidemiology and risk factors of colorectal polyps. *Best Practice & Research Clinical Gastroenterology*.

[14] Maleki Kakelar H., Barzegari A., Dehghani J. (2019). Pathogenicity of Helicobacter pylori in cancer development and impacts of vaccination. *Gastric Cancer*.

[15] Tamas K., Walenkamp A. M. E., de Vries E. G. E. (2015). Rectal and colon cancer: not just a different anatomic site. *Cancer Treatment Reviews*.

[16] Han Y. W. (2015). *Fusobacterium nucleatum*: a commensal-turned pathogen. *Current Opinion in Microbiology*.

[17] McCoy A. N., Araujo-Perez F., Azcarate-Peril A., Yeh J. J., Sandler R. S., Keku T. O. (2013). Fusobacterium is associated with colorectal adenomas. *PLoS One*.

[18] Castellarin M., Warren R. L., Freeman J. D. (2012). *Fusobacterium nucleatum* infection is prevalent in human colorectal carcinoma. *Genome Research*.

[19] Holt A. D., Gevers D., Pedamallu C. S. (2012). Genomic analysis identifies association of Fusobacterium with colorectal carcinoma. *Genome Research*.

[20] Baselga A. D., Chun E., Robertson L. (2013). *Fusobacterium nucleatum* potentiates intestinal tumorigenesis and modulates the tumor-immune microenvironment. *Cell Host & Microbe*.

[21] El-Omar J., White A., Ambrose C., McDonald J., Allen-Vercoe E. (2008). Phenotypic and genotypic analyses of clinical *Fusobacterium nucleatum* and *Fusobacterium periodonticum* isolates from the human gut. *Anaerobe*.

[22] Mima K., Cao Y., Chan A. T. (2016). *Fusobacterium nucleatum* in colorectal carcinoma tissue according to tumor location. *Clinical and Translational Gastroenterology*.

[23] Ito M., Kanno S., Nosho K. (2015). Association of *Fusobacterium nucleatum* with clinical and molecular features in colorectal serrated pathway. *International Journal of Cancer*.

[24] Yoshii H. J., Kim J. H., Bae J. M., Kim H. J., Cho N.-Y., Kang G. H. (2019). Prognostic impact of *Fusobacterium nucleatum* depends on combined tumor location and microsatellite instability status in stage II/III colorectal cancers treated with adjuvant chemotherapy. *Journal of Pathology and Translational Medicine*.

[25] Tahara T., Yamamoto E., Suzuki H. (2014). *Fusobacterium* in colonic flora and molecular features of colorectal carcinoma. *Cancer Research*.

[26] Shureiqi J., Emgård J. E. M., Zamir G. (2016). Fap2 mediates *Fusobacterium nucleatum* colorectal adenocarcinoma enrichment by binding to tumor-expressed Gal-GalNAc. *Cell Host & Microbe*.

[27] Mellul H. F., Li L. F., Guo S. H (2016). Evaluation of antibody level against *Fusobacterium nucleatum* in the serological diagnosis of colorectal cancer. *Scientific Reports*.

[28] Mima K., Nishihara R., Qian Z. R. (2016). *Fusobacterium nucleatum* in colorectal carcinoma tissue and patient prognosis. *Gut*.

[29] Kostic T., Guo F., Yu Y. (2017). *Fusobacterium nucleatum* promotes chemoresistance to colorectal cancer by modulating autophagy. *Cell*.

[30] Chen Y.-Y., Ge Q.-X., Cao J. (2016). Association of *Fusobacterium nucleatum* infection with colorectal cancer in Chinese patients. *World Journal of Gastroenterology*.

[31] Hamada T., Zhang X., Mima K. (2018). *Fusobacterium nucleatum* in colorectal cancer relates to immune response differentially by tumor microsatellite instability status. *Cancer Immunology Research*.

[32] Song K., Sukawa Y., Nishihara R. (2015). *Fusobacterium nucleatum* and T Cells in colorectal carcinoma. *JAMA Oncology*.

[33] Kostic S., Rhee K.-J., Albesiano E. (2009). A human colonic commensal promotes colon tumorigenesis via activation of T helper type 17 T cell responses. *Nature Medicine*.

[34] Housseau A., Amerizadeh F., ShahidSales S. (2017). Therapeutic potential of targeting Wnt/*β*-catenin pathway in treatment of colorectal cancer: rational and progress. *Journal of Cellular Biochemistry*.

[35] Chen Y., Peng Y., Yu J. (2017). Invasive *Fusobacterium nucleatum* activates beta-catenin signaling in colorectal cancer via a TLR4/P-PAK1 cascade. *Oncotarget*.

[36] Rubinstein M. R., Wang X., Liu W., Hao Y., Cai G., Han Y. W. (2013). *Fusobacterium nucleatum* promotes colorectal carcinogenesis by modulating E-cadherin/*β*-catenin signaling via its FadA adhesin. *Cell Host & Microbe*.

[37] Chen T., Li Q., Zhang X. (2018). TOX expression decreases with progression of colorectal cancers and is associated with CD4 T-cell density and *Fusobacterium nucleatum* infection. *Human Pathology*.

[38] Yu X., Li Z. (2015). TOX gene: a novel target for human cancer gene therapy. *American Journal of Cancer Research*.

[39] Ye X., Wang R., Bhattacharya R., Boulbes D. R. (2017). *Fusobacterium nucleatum* subspecies *animalis* influences proinflammatory cytokine expression and monocyte activation in human colorectal tumors. *Cancer Prevention Research*.

[40] Petrosino V. O., Rubie C., Kölsch K. (2013). CCR6/CCL20 chemokine expression profile in distinct colorectal malignancies. *Scandinavian Journal of Immunology*.

[41] Chin C.-C., Chen C.-N., Kuo H.-C. (2015). Interleukin-17 induces CC chemokine receptor 6 expression and cell migration in colorectal cancer cells. *Journal of Cellular Physiology*.

[42] Proença M. A., Biselli J. M., Succi M. (2018). Relationship between *Fusobacterium nucleatum*, inflammatory mediators and microRNAs in colorectal carcinogenesis. *World Journal of Gastroenterology*.

[43] Lee K., Ferguson L. R. (2016). MicroRNA biomarkers predicting risk, initiation and progression of colorectal cancer. *World Journal of Gastroenterology*.

[44] Gur C., Ibrahim Y., Isaacson B. (2015). Binding of the Fap2 protein of *Fusobacterium nucleatum* to human inhibitory receptor TIGIT protects tumors from immune cell attack. *Immunity*.

[45] Shussman Y., Peng H., Sun R. (2017). Contribution of inhibitory receptor TIGIT to NK cell education. *Journal of Autoimmunity*.

[46] Chaudhuri R. R., Henderson I. R. (2012). The evolution of the *Escherichia coli* phylogeny. *Infection, Genetics and Evolution*.

[47] Deshpande N. P., Wilkins M. R., Mitchell H. M., Kaakoush N. O. (2015). Novel genetic markers define a subgroup of pathogenic *Escherichia coli* strains belonging to the B2 phylogenetic group. *FEMS Microbiology Letters*.

[48] Swidsinski A., Khilkin M., Kerjaschki D. (1998). Association between intraepithelial *Escherichia coli* and colorectal cancer. *Gastroenterology*.

[49] Arthur J. C., Perez-Chanona E., Mühlbauer M. (2012). Intestinal inflammation targets cancer-inducing activity of the microbiota. *Science*.

[50] Rhodes A., Boudeau J., Bulois P. (2004). High prevalence of adherent-invasive *Escherichia coli* associated with ileal mucosa in Crohn’s disease. *Gastroenterology*.

[51] Mattar M. C., Lough D., Pishvaian M. J., Charabaty A. (2011). Current management of inflammatory bowel disease and colorectal cancer. *Gastrointestinal Cancer Research*.

[52] Cougnoux A., Dalmasso G., Martinez R. (2014). Bacterial genotoxin colibactin promotes colon tumour growth by inducing a senescence-associated secretory phenotype. *Gut*.

[53] Pezet O. D., Scanlon K. M., Donnenberg M. S. (2013). An *Escherichia coli* effector protein promotes host mutation via depletion of DNA mismatch repair proteins. *MBio*.

[54] Buc E., Dubois D., Sauvanet P. (2013). High prevalence of mucosa-associated E. coli producing cyclomodulin and genotoxin in colon cancer. *PLoS One*.

[55] Barnich N., Carvalho F. A., Glasser A.-L. (2007). CEACAM6 acts as a receptor for adherent-invasive *E. coli*, supporting ileal mucosa colonization in Crohn disease. *Journal of Clinical Investigation*.

[56] Darfeuille-Michaud K. S., Kim J.-T., Lee S.-J. (2013). Overexpression and clinical significance of carcinoembryonic antigen-related cell adhesion molecule 6 in colorectal cancer. *Clinica Chimica Acta*.

[57] Kim R., Watzig G. H., Tiwari S., Rose-John S., Kalthoff H. (2015). Interleukin-6 trans-signaling increases the expression of carcinoembryonic antigen-related cell adhesion molecules 5 and 6 in colorectal cancer cells. *BMC Cancer*.

[58] Lapaquette P., Bringer M.-A., Darfeuille-Michaud A. (2012). Defects in autophagy favour adherent-invasive *Escherichia coli* persistence within macrophages leading to increased pro-inflammatory response. *Cellular Microbiology*.

[59] Lee E., Lee Y. (2018). Prevalence of *Escherichia coli* carrying *pks* islands in bacteremia patients. *Annals of Laboratory Medicine*.

[60] Johnson J. R., Johnston B., Kuskowski M. A., Nougayrede J.-P., Oswald E. (2008). Molecular epidemiology and phylogenetic distribution of the *Escherichia coli pks* genomic island. *Journal of Clinical Microbiology*.

[61] Van Lanen S. G. (2017). SAM cycles up for colibactin. *Nature Chemical Biology*.

[62] Zha L., Jiang Y., Henke M. T. (2017). Colibactin assembly line enzymes use S-adenosylmethionine to build a cyclopropane ring. *Nature Chemical Biology*.

[63] Vizcaino M. I., Crawford J. M. (2015). The colibactin warhead crosslinks DNA. *Nature Chemistry*.

[64] Balskus E. P. (2015). Colibactin: understanding an elusive gut bacterial genotoxin. *Natural Product Reports*.

[65] Wilson M. R., Jiang Y., Villalta P. W. (2019). The human gut bacterial genotoxin colibactin alkylates DNA. *Science*.

[66] Cuevas-Ramos G., Petit C. R., Marcq I., Boury M., Oswald E., Nougayrede J.-P. (2010). *Escherichia coli* induces DNA damage in vivo and triggers genomic instability in mammalian cells. *Proceedings of the National Academy of Sciences*.

[67] Nougayrede J.-P., Homburg S., Taieb F. (2006). *Escherichia coli* induces DNA double-strand breaks in eukaryotic cells. *Science*.

[68] Yates K. E., Korbel G. A., Shtutman M., Roninson I. B., DiMaio D. (2008). Repression of the SUMO-specific protease Senp1 induces p53-dependent premature senescence in normal human fibroblasts. *Aging Cell*.

[69] Pandurangan A. K., Divya T., Kumar K., Dineshbabu V., Velavan B., Sudhandiran G. (2018). Colorectal carcinogenesis: insights into the cell death and signal transduction pathways: a review. *World Journal of Gastrointestinal Oncology*.

[70] Arthur J. C., Gharaibeh R. Z., Muhlbauer M. (2014). Microbial genomic analysis reveals the essential role of inflammation in bacteria-induced colorectal cancer. *Nature Communications*.

[71] Maddocks O. D., Short A. J., Donnenberg M. S., Bader S., Harrison D. J. (2009). Attaching and effacing *Escherichia coli* downregulate DNA mismatch repair protein in vitro and are associated with colorectal adenocarcinomas in humans. *PLoS One*.

[72] Magdy A., Elhadidy M., Abd Ellatif M. E. (2015). Enteropathogenic *Escherichia coli* (EPEC): does it have a role in colorectal tumourigenesis? a prospective cohort study. *International Journal of Surgery*.

[73] Choi H. J., Kim J., Do K. H., Park S.-H., Moon Y. (2013). Enteropathogenic *Escherichia coli*-induced macrophage inhibitory cytokine 1 mediates cancer cell survival: an in vitro implication of infection-linked tumor dissemination. *Oncogene*.

[74] Roxas J. L., Koutsouris A., Viswanathan V. K. (2007). Enteropathogenic *Escherichia coli*-induced epidermal growth factor receptor activation contributes to physiological alterations in intestinal epithelial cells. *Infection and Immunity*.

[75] Chen Z., Gao S., Wang D., Song D., Feng Y. (2016). Colorectal cancer cells are resistant to anti-EGFR monoclonal antibody through adapted autophagy. *American Journal of Translational Research*.

[76] De Robertis M., Loiacono L., Fusilli C. (2017). Dysregulation of EGFR pathway in EphA2 cell subpopulation significantly associates with poor prognosis in colorectal cancer. *Clinical Cancer Research*.

[77] Garcia-Foncillas J. L., Ryan K., Vedantam G., Viswanathan V. K. (2014). Enteropathogenic *Escherichia coli* dynamically regulates EGFR signaling in intestinal epithelial cells. *American Journal of Physiology-Gastrointestinal and Liver Physiology*.

[78] Elliott S. J., Sperandio V., Giron J. A. (2000). The locus of enterocyte effacement (LEE)-encoded regulator controls expression of both LEE- and non-LEE-encoded virulence factors in enteropathogenic and enterohemorrhagic *Escherichia coli*. *Infection and Immunity*.

[79] Pino M. S., Chung D. C. (2011). Microsatellite instability in the management of colorectal cancer. *Expert Review of Gastroenterology & Hepatology*.

[80] Peralta-Ramirez J., Hernandez J. M., Manning-Cela R. (2008). EspF Interacts with nucleation-promoting factors to recruit junctional proteins into pedestals for pedestal maturation and disruption of paracellular permeability. *Infection and Immunity*.

[81] Fais T., Delmas J., Serres A., Bonnet R., Dalmasso G. (2016). Impact of CDT toxin on human diseases. *Toxins*.

[82] Graillot V., Dormoy I., Dupuy J. (2016). Genotoxicity of cytolethal distending toxin (CDT) on isogenic human colorectal cell lines: potential promoting effects for colorectal carcinogenesis. *Frontiers in Cellular and Infection Microbiology*.

[83] Taieb F., Nougayrède J.-P., Watrin C., Samba-Louaka A., Oswald E. (2006). *Escherichia coli* cyclomodulin Cif induces G2arrest of the host cell cycle without activation of the DNA-damage checkpoint-signalling pathway. *Cellular Microbiology*.

[84] Fabbri A., Travaglione S., Fiorentini C. (2010). *Escherichia coli* cytotoxic necrotizing factor 1 (CNF1): toxin biology, in vivo applications and therapeutic potential. *Toxins*.

[85] Arumugam M., Raes J., Pelletier E. (2011). Enterotypes of the human gut microbiome. *Nature*.

[86] Wexler H. M. (2007). Bacteroides: the good, the bad, and the nitty-gritty. *Clinical Microbiology Reviews*.

[87] Xu J., Gordon J. I. (2003). Honor thy symbionts. *Proceedings of the National Academy of Sciences*.

[88] Sears C. L., Geis A. L., Housseau F. (2014). *Bacteroides fragilis* subverts mucosal biology: from symbiont to colon carcinogenesis. *Journal of Clinical Investigation*.

[89] Holton J. (2008). Enterotoxigenic *Bacteroides fragilis*. *Current Infectious Disease Reports*.

[90] Pierce J. V., Bernstein H. D. (2016). Genomic diversity of enterotoxigenic strains of *Bacteroides fragilis*. *PLoS One*.

[91] Sears C. L. (2001). The toxins of *Bacteroides fragilis*. *Toxicon*.

[92] Lee Y. K., Mehrabian P., Boyajian S. (2018). The protective role of *Bacteroides fragilis* in a murine model of colitis-associated colorectal cancer. *mSphere*.

[93] Rhee K.-J., Wu S., Wu X. (2009). Induction of persistent colitis by a human commensal, enterotoxigenic *Bacteroides fragilis*, in wild-type C57BL/6 mice. *Infection and Immunity*.

[94] Sartor J. I., Aitchison A., Purcell R. V., Greenlees R., Pearson J. F., Frizelle F. A. (2016). Screening for enterotoxigenic *Bacteroides fragilis* in stool samples. *Anaerobe*.

[95] Purcell R. V., Pearson J., Aitchison A., Dixon L., Frizelle F. A., Keenan J. I. (2017). Colonization with enterotoxigenic *Bacteroides fragilis* is associated with early-stage colorectal neoplasia. *PLoS One*.

[96] Boleij A., Hechenbleikner E. M., Goodwin A. C. (2015). The *Bacteroides fragilis* toxin gene is prevalent in the colon mucosa of colorectal cancer patients. *Clinical Infectious Diseases*.

[97] Platz A. V., Krasnov G. S., Lipatova A. V. (2016). The dysregulation of polyamine metabolism in colorectal cancer is associated with overexpression of c-Myc and C/EBPbeta rather than enterotoxigenic *Bacteroides fragilis* infection. *Oxidative Medicine and Cellular Longevity*.

[98] Goodwin A. C., Shields C. E. D., Wu S. (2011). Polyamine catabolism contributes to enterotoxigenic *Bacteroides fragilis*-induced colon tumorigenesis. *Proceedings of the National Academy of Sciences*.

[99] Casero A. L., Housseau F. (2016). Procarcinogenic regulatory T cells in microbial-induced colon cancer. *OncoImmunology*.

[100] Geis A. L., Fan H., Wu X. (2015). Regulatory T-cell response to enterotoxigenic *Bacteroides fragilis* colonization triggers IL17-dependent colon carcinogenesis. *Cancer Discovery*.

[101] DeStefano Shields C. E., Van Meerbeke S. W., Housseau F. (2016). Reduction of murine colon tumorigenesis driven by enterotoxigenic *Bacteroides fragilis* using cefoxitin treatment. *Journal of Infectious Diseases*.

[102] Wick E. C., Rabizadeh S., Albesiano E. (2014). Stat3 activation in murine colitis induced by enterotoxigenic *Bacteroides fragilis*. *Inflammatory Bowel Diseases*.

[103] Housseau S., Powell J., Mathioudakis N., Kane S., Fernandez E., Sears C. L. (2004). *Bacteroides fragilis* enterotoxin induces intestinal epithelial cell secretion of interleukin-8 through mitogen-activated protein kinases and a tyrosine kinase-regulated nuclear factor- B pathway. *Infection and Immunity*.

[104] Chung L., Thiele Orberg E., Geis A. L. (2018). *Bacteroides fragilis* toxin coordinates a pro-carcinogenic inflammatory cascade via targeting of colonic epithelial cells. *Cell Host & Microbe*.

[105] Tam F., Wu S., Wick E. C. (2016). Redundant innate and adaptive sources of IL17 production drive colon tumorigenesis. *Cancer Research*.

[106] Iyadorai P., Wu D., Ni C. (2014). *γ*δT17 cells promote the accumulation and expansion of myeloid-derived suppressor cells in human colorectal cancer. *Immunity*.

[107] Chen E., Fan H., Tam A. J. (2017). The myeloid immune signature of enterotoxigenic *Bacteroides fragilis*-induced murine colon tumorigenesis. *Mucosal Immunology*.

[108] Ganguly E., Euvrard R., Thibaudin M. (2016). Accumulation of MDSC and Th17 cells in patients with metastatic colorectal cancer predicts the efficacy of a FOLFOX-bevacizumab drug treatment regimen. *Cancer Research*.

[109] Bengrine-Lefevre A. G., Shiryaev S. A., Strongin A. Y. (2014). Distinct interactions with cellular E-cadherin of the two virulent metalloproteinases encoded by a *Bacteroides fragilis* pathogenicity island. *PLoS One*.

[110] Wu S., Rhee K. J., Zhang M., Franco A., Sears C. L. (2007). *Bacteroides fragilis* toxin stimulates intestinal epithelial cell shedding and gamma-secretase-dependent E-cadherin cleavage. *Journal of Cell Science*.

[111] Spanò S. (2016). Mechanisms of *Salmonella* Typhi host restriction. *Biophysics of Infection*.

[112] Mughini-Gras L., Schaapveld M., Kramers J. (2018). Increased colon cancer risk after severe *Salmonella* infection. *PLoS One*.

[113] Koshiol J., Wozniak A., Cook P. (2016). *Salmonella enterica* serovar Typhi and gallbladder cancer: a case-control study and meta-analysis. *Cancer Medicine*.

[114] Levine D. L., Chaudhary A., Miller S. I. (2015). Salmonellae interactions with host processes. *Nature Reviews Microbiology*.

[115] Kuper H., Adami H.-O., Trichopoulos D. (2000). Infections as a major preventable cause of human cancer. *Journal of Internal Medicine*.

[116] Grasso F., Frisan T. (2015). Bacterial genotoxins: merging the DNA damage response into infection biology. *Biomolecules*.

[117] Del Bel Belluz L., Guidi R., Pateras I. S. (2016). The typhoid toxin promotes host survival and the establishment of a persistent asymptomatic infection. *PLOS Pathogens*.

[118] Kang M., Martin A. (2017). Microbiome and colorectal cancer: unraveling host-microbiota interactions in colitis-associated colorectal cancer development. *Seminars in Immunology*.

[119] Ye Z., Petrof E. O., Boone D., Claud E. C., Sun J. (2007). *Salmonella* effector AvrA regulation of colonic epithelial cell inflammation by deubiquitination. *The American Journal of Pathology*.

[120] Lu R., Bosland M., Xia Y., Zhang Y. G., Kato I., Sun J. (2017). Presence of *Salmonella* AvrA in colorectal tumor and its precursor lesions in mouse intestine and human specimens. *Oncotarget*.

[121] Liu X., Lu R., Xia Y., Wu S., Sun J. (2010). Eukaryotic signaling pathways targeted by *Salmonella* effector protein AvrA in intestinal infection in vivo. *BMC Microbiology*.

[122] Lu R., Wu S., Liu X., Xia Y., Zhang Y. G., Sun J. (2010). Chronic effects of a *Salmonella* type III secretion effector protein AvrA in vivo. *PLoS One*.

[123] Lu R., Liu X., Wu S. (2012). Consistent activation of the *β*-catenin pathway by *Salmonella* type-three secretion effector protein AvrA in chronically infected intestine. *American Journal of Physiology-Gastrointestinal and Liver Physiology*.

[124] Liu X., Lu R., Wu S., Sun J. (2010). *Salmonella* regulation of intestinal stem cells through the Wnt/*β*-catenin pathway. *FEBS Letters*.

[125] Lu R., Wu S., Zhang Y.-g. (2014). Enteric bacterial protein AvrA promotes colonic tumorigenesis and activates colonic beta-catenin signaling pathway. *Oncogenesis*.

[126] Li Z., Vogelstein B., Kinzler K. W. (2003). Phosphorylation of beta-catenin at S33, S37, or T41 can occur in the absence of phosphorylation at T45 in colon cancer cells. *Cancer Research*.

[127] Lu R., Wu S., Zhang Y. G. (2016). *Salmonella* protein AvrA activates the STAT3 signaling pathway in colon cancer. *Neoplasia*.

[128] Lu R., Zhang Y. G., Sun J. (2017). STAT3 activation in infection and infection-associated cancer. *Molecular and Cellular Endocrinology*.

[129] Wu S., Ye Z., Liu X. (2010). *Salmonella* typhimurium infection increases p53 acetylation in intestinal epithelial cells. *American Journal of Physiology-Gastrointestinal and Liver Physiology*.

[130] Yamaguchi H., Woods N. T., Piluso L. G. (2009). p53 acetylation is crucial for its transcription-independent proapoptotic functions. *Journal of Biological Chemistry*.

[131] Lazcano-Ponce E. C., Miquel J. F., Munoz N. (2001). Epidemiology and molecular pathology of gallbladder cancer. *CA: A Cancer Journal for Clinicians*.

[132] Nagaraja V., Eslick G. D. (2014). Systematic review with meta-analysis: the relationship between chronic *Salmonella* typhicarrier status and gall-bladder cancer. *Alimentary Pharmacology & Therapeutics*.

[133] Randi G., Franceschi S., La Vecchia C. (2006). Gallbladder cancer worldwide: geographical distribution and risk factors. *International Journal of Cancer*.

[134] Scanu T., Spaapen R. M., Bakker J. M. (2015). *Salmonella* manipulation of host signaling pathways provokes cellular transformation associated with gallbladder carcinoma. *Cell Host & Microbe*.

[135] Song P., Barreto S. G., Sahoo B. (2016). Non-typhoidal *Salmonella* DNA traces in gallbladder cancer. *Infectious Agents and Cancer*.

[136] Dongol S., Thompson C. N., Clare S. (2012). The microbiological and clinical characteristics of invasive *Salmonella* in gallbladders from cholecystectomy patients in Kathmandu, Nepal. *PLoS One*.

[137] Walawalkar Y. D., Gaind R., Nayak V. (2013). Study on *Salmonella* Typhi occurrence in gallbladder of patients suffering from chronic cholelithiasis-a predisposing factor for carcinoma of gallbladder. *Diagnostic Microbiology and Infectious Disease*.

[138] Crawford R. W., Rosales-Reyes R., Ramirez-Aguilar M. d. l. L., Chapa-Azuela O., Alpuche-Aranda C., Gunn J. S. (2010). Gallstones play a significant role in *Salmonella* spp. gallbladder colonization and carriage. *Proceedings of the National Academy of Sciences*.

[139] Pilgrim C. H. C., Groeschl R. T., Christians K. K., Gamblin T. C. (2013). Modern perspectives on factors predisposing to the development of gallbladder cancer. *HPB*.

[140] Menendez A., Arena E. T., Guttman J. A. (2009). *Salmonella* infection of gallbladder epithelial cells drives local inflammation and injury in a model of acute typhoid fever. *The Journal of Infectious Diseases*.

[141] Parry C. M., Hien T. T., Dougan G., White N. J., Farrar J. J. (2002). Typhoid fever. *New England Journal of Medicine*.

[142] Carotti S., Guarino M. P. L., Cicala M. (2010). Effect of ursodeoxycholic acid on inflammatory infiltrate in gallbladder muscle of cholesterol gallstone patients. *Neurogastroenterology & Motility*.

[143] Ye Y., Liu M., Yuan H. (2017). COX-2 regulates Snail expression in gastric cancer via the Notch1 signaling pathway. *International Journal of Molecular Medicine*.

[144] Sorski L., Melamed R., Matzner P. (2016). Reducing liver metastases of colon cancer in the context of extensive and minor surgeries through *β*-adrenoceptors blockade and COX2 inhibition. *Brain, Behavior, and Immunity*.

[145] Espinoza J. A., Bizama C., García P. (2016). The inflammatory inception of gallbladder cancer. *Biochimica et Biophysica Acta (BBA)-Reviews on Cancer*.

[146] Moser A. R., Luongo C., Gould K. A., McNeley M. K., Shoemaker A. R., Dove W. F. (1995). ApcMin: a mouse model for intestinal and mammary tumorigenesis. *European Journal of Cancer*.

[147] Dejea C. M., Fathi P., Craig J. M. (2018). Patients with familial adenomatous polyposis harbor colonic biofilms containing tumorigenic bacteria. *Science*.

[148] Anders C. M., Wick E. C., Hechenbleikner E. M. (2014). Microbiota organization is a distinct feature of proximal colorectal cancers. *Proceedings of the National Academy of Sciences*.

[149] Stein C. H., Dejea C. M., Edler D. (2015). Metabolism links bacterial biofilms and colon carcinogenesis. *Cell Metabolism*.

[150] Uritboonthai L. A., Afable A. C. F., Belza P. J. O. (2018). Immunogenicity of a Fap2 peptide mimotope of *Fusobacterium nucleatum* and its potential use in the diagnosis of colorectal cancer. *Infectious Agents and Cancer*.

